# Fermentation and Quality Characteristics of Yogurt Treated with *Bifidobacterium longum*

**DOI:** 10.3390/nu15153490

**Published:** 2023-08-07

**Authors:** Jang Keun Son, Yeon Jae Jo, Yun Jo Jung, Youn Ri Lee, Junsoo Lee, Heon Sang Jeong

**Affiliations:** 1 Department of Food Science & Biotechnology, Chungbuk National University, Cheongju-si 28644, Republic of Korea; wkdrms0603@naver.com (J.K.S.); yjcho6522@naver.com (Y.J.J.); yunjo96@naver.com (Y.J.J.); junsoo@chungbuk.ac.kr (J.L.); 2 Department of Food and Nutrition, Daejeon Health Sciences College, Daejeon 34504, Republic of Korea; leeyounri@hit.ac.kr

**Keywords:** *Bifidobacterium longum*, lactose, yogurt, lactic acid bacteria, quality

## Abstract

The fermentation and quality characteristics of yogurt were investigated according to the inoculation concentration of *Bifidobacterium longum*. The total sugar content of yogurt decreased as the fermentation time increased, and with an increased concentration of *B. longum* treatment the fermentation time decreased rapidly. As fermentation progressed, the lactose content decreased rapidly at the beginning and gradually decreased as the pH decreased. Depending on the *B. longum* treatment concentration, the lactose content varied from 0.29 ± 0.01 to 0.47 ± 0.01% and was 0.5% or less in all experimental groups. The experimental group inoculated with 0.0015% of *B. longum* displayed the best results in all categories, including pH, total acidity, lactic acid content, solid non-fat content, and total lactic acid bacteria count, which are factors that determine the quality of yogurt. In summary, the experimental group inoculated with 0.0015% of *B. longum* was determined to be the highest quality yogurt.

## 1. Introduction

In addition to its anti-cancer and blood cholesterol-lowering effects, yogurt is known for its high nutritional and hydration levels, ability to reduce lactose intolerance, increase beneficial bacteria such as bifidobacteria, inhibit growth of intestinal pathogenic bacteria, and improve constipation [1]. *Lactobacillus bulgaricus*, *L. acidophilus*, *L. casei*, and *Streptococcus salivarius* subsp. *thermophilus* are the major lactic acid bacteria used in yogurt production [2] Given its improved nutrition, digestibility, unique flavor, and various physiological functions, the demand for yogurt is steadily increasing worldwide [3]. Because the demand for yogurt and other milk products with high milk solid content and lactic acid bacteria has been steadily increasing for several years, liquid yogurt has been the main type of yogurt popular in Korea [4]. The Codex standards for fermented milk state that the number of starter culture bacteria should be at least 10^7^ CFU g^−1^ throughout its shelf life and the minimum numbers required for health benefits should be at least 10^6^ CFU g^−1^ [5]. In Korea, it can be called yogurt only when it contains more than 100 million lactic acid bacteria and has a non-solid content of 8% or more [6].

Yogurt fermented with *Lactobacillus bulgaricus*, *Streptococcus salivarius* subsp. *thermophilus*, and *Bifidobacterium longum* together has higher viscosity than separately fermented, respectively. Additionally, specific exopolysaccharide (EPS) producing bifidobacterial could improve the quality of fermented milk products, including physicochemical properties, as well as endowing products with health benefits [7].

Lactose is primarily obtained by consuming dairy products. In general, milk contains approximately 5% lactose, whereas yogurt has a lactose content of approximately 3.5%. However, more than 70% of the global population is genetically incapable of digesting lactose [8]. *Bifidobacterium longum* is a Gram-positive, catalase-negative, rod-shaped bacterium present in the human gastrointestinal tract and it is one of the 32 species belonging to the genus Bifidobacterium [9]. *B. longum* is non-pathogenic and is often added to food products. *B. longum* is the most common species of Bifidobacteria, it has better growth properties in milk than other species of this genus but is usually used as supplements [10]. Many studies have been conducted on the production of lactose-free dairy products; however, little research has been conducted on lactose-free yogurt.

Therefore, this study examined the quality and fermentation characteristics of lactose-free yogurt fermented directly by *B. longum,* rather than its use as a supplement. Additionally, yogurt manufacturing conditions were established according to the optimal *B. longum* inoculation conditions with lactase.

## 2. Materials and Methods

### 2.1. Materials

Raw milk, sterilized milk, *Bifidobacterium longum* (Bifidobacteriul longum KACC 91563), lactase (MAXILACT LGI 5000), and yogurt lactic acid bacteria (Lyofast SAB 440 B, SACCO) used in this experiment were provided by Cheongwon Natural Land located in Cheongju-si, Republic of Korea.

### 2.2. Manufacture of Yogurt

To prepare the yogurt, 0.032% lactic acid bacteria (Lyofast SAB 440 B, SACCO, Cadorago, Italy) containing *Streptococcus salivarius* subsp. *thermophilus*, *Lactobacillus acidophilus*, *Bifidobacterium animalis* ssp., and 15% lactase were added to milk based on its volume. To examine the effect of *Bifidobacterium longum* (KACC91563), it was inoculated at concentrations of 0.001, 0.00125, and 0.0015% by volume of milk, and the unadded sample was used as the control. Cells were allowed to ferment in an incubator at 37 °C for 8 h. Subsequently, the mixture was refrigerated at 4 °C for 15 h and used in the experiments.

### 2.3. Measurement of Total Sugar Content

The total sugar content of the yogurt was measured using the phenol-sulfuric acid method [11]. The sample was diluted to an appropriate ratio, 0.5 mL of a 5% phenol (Shinyo Pure Chemicals Co., Ltd., Osaka, Japan) solution was added to 1 mL of the diluent, 2.5 mL of 95% sulfuric acid (Daejung Chemicals and Metals Co., Ltd., Siheung, Republic of Korea) was added, and the mixture was left at room temperature for 30 min. The absorbance was measured at 470 nm using an Epoch microplate spectrophotometer (Biotek Instruments Inc., Winooski, VT, USA). The calibration curve was prepared using glucose as the standard and expressed as equivalent milligrams of glucose per milliliter.

### 2.4. Measurement of Reducing Sugar Content

The reduced sugar content of the yogurt was measured using the DNS method [12] after diluting the sample to an appropriate ratio. After adding 0.4 mL of DNS reagent to 0.2 mL of the sample, heating it in boiling water at 100 °C for 5 min, cooling it rapidly, and adding 1.8 mL of distilled water, the sugar content was measured using an Epoch microplate spectrophotometer (Biotek Instruments Inc., VT, USA). The absorbance was measured at 525 nm. For the standard, a calibration curve was prepared using glucose and expressed as milligrams of glucose equivalents per milliliter.

### 2.5. Measurement of Lactose Content

The lactose content of yogurt was analyzed by modifying the method described by Kim et al. [13]. Subsequently, 49 mL of 70% acetonitrile was added to 1 mL of the sample, diluted 50 times, filtered through a 0.2 μm membrane filter, and analyzed using HPLC (Jasco System, Tokyo, Japan). A Luna 5 μm NH-2 100Å column (4.6 × 250 mm ID, Phenomenex, Torrance, CA, USA) was used. Acetonitrile water (80:20, *v*/*v*) was used as the mobile phase. An ELSD detector was used (Waters 2420, Waters, Milford, MA, USA). The flow rate was 1 mL/min and the injection volume was 20 μL. Lactose (Sigma-Aldrich, St. Louis, MO, USA) was used as the standard.

### 2.6. Measurement of pH, Total Acidity, and Lactic Acid Content

The pH of the yogurt was measured using a pH meter (Orion 4 Star; Thermo Scientific, Beverly, MA, USA). The total acidity of the yogurt was determined as the amount of standardized 0.1 N NaOH required for neutralization using 1% phenolphthalein as an indicator. It was expressed as lactic acid (%) content [14]. The lactic acid content of the yogurt was analyzed by modifying the method described by Hwang et al. [15]. The yogurt was diluted to an appropriate concentration, filtered through a 0.45 μm membrane filter, and analyzed by HPLC (YL9120 system, Younglin, Anyang, Republic of Korea). The column used was a YMC-Triart C18 column (4.6 × 250 mm ID, YMC Co., Ltd., Kyoto, Japan). The absorbance was detected at 215 nm using a UV detector. The mobile phase was a 20 mM potassium phosphate-buffered solution (pH 2.8). The flow rate was 0.6 mL/min and the injection volume was 20 μL. Lactic acid was used as a standard.

### 2.7. Measurement of Viscosity

The viscosity of the yogurt during fermentation was measured at 37 °C by the viscometer (DV-Ⅱ+ Pro, Ametek Brookfield, Middleboro, MA, USA) with 1, 2, 3, 4, and 5 spindles at 200 rpm. All values were measured five times.

### 2.8. Measurement of Solids Non-Fat

Solids non-fat content was measured using the Food Code analysis method [16]. Next, 15 g of purified sea sand was placed with a small glass rod in a weighing tube with a base diameter of 5 cm or more, and it was dried in a dryer at 98–100 °C until a constant weight was achieved. Next, we accurately weighed about 5 g of the test sample, placed it in a weighing tube, and stirred the contents in a water bath while mixing. After most of the moisture was evaporated by heating, the sample was transferred to a dryer and dried until a constant weight was obtained to determine the amount of dry matter. The percentages of crude fat and sugar (sucrose, fructose, and glucose) were subtracted from the percentage of dry matter to obtain the percentage of solids content.

### 2.9. Measurement of Total Lactic Acid Bacteria Cell Number

The total lactic acid bacteria cell number was confirmed by the number of colonies after incubating for 48 h at 37 °C in anaerobic condition and dispensing 1 mL of a sample diluted to 10^6^–10^8^ using a Petrifilm medium (Lactic acid bacteria count plate, 3M, Maplewood, NJ, USA) for lactic acid bacteria.

### 2.10. Statistical Analysis

All analyses were repeated three times and expressed as mean ± SD. Statistical analyses were performed using the SPSS statistical program (Statistical Package for the Social Science, ver. 12.0, IBM SPSS Statistics, Chicago, IL, USA), which was used to calculate the mean and standard deviation of each treatment group. Subsequently, a one-way ANOVA test and Duncan’s multiple range test was used to test the significance.

## 3. Results and Discussions

### 3.1. Total Sugar and Reducing Sugar Content

Changes in the total sugar content of the yogurt according to the inoculation concentration of *B. longum* and fermentation time are presented in Table 1. With increasing fermentation time and inoculation concentration of *B. longum*, the total sugar content decreased. After 4 h of fermentation, the total sugar content was 78.63 mg/mL in the control group and 76.14 mg/mL in the 0.0015% inoculum of *B. longum*, and after 8 h of fermentation, the total sugar content was 72.62 and 69.91 mg/mL, respectively. These results were similar to those reported by Nguyen and Hwang [17]. As displayed in Table 1, the reducing sugar content increased during the early fermentation stage and then decreased subsequently. The increase in reducing sugars in the early fermentation stage is attributed to the high lactase activity and the decomposition of lactose into glucose and galactose [18]. After 4 h of fermentation, the reducing sugar content decreased because of the increased conversion of reducing sugars into acids compared with the decomposition of lactose. In the case of the control group, it increased from 44.33 mg/mL to 53.89 mg/mL after 4 h of fermentation and then decreased to 50.63 mg/mL at the end of fermentation. As the *B. longum* inoculum concentration increased, the reducing sugar content decreased. The group inoculated with 0.0015% of *B. longum* displayed an increase from 44.12 mg/mL before fermentation to 50.19 mg/mL after 4 h of fermentation and decreased to 46.97 mg/mL after 8 h of fermentation, and these results were similar to those reported in a study by Kim et al. [13]. As fermentation proceeds, sugar is converted to acid and its content decreases. Therefore, the change of total and reducing sugar contents in this experiment indicates that the higher concentration of *B. longum* fermented more effectively.

### 3.2. Lactose Content

Changes in the lactose content of yogurt according to the inoculation concentration and fermentation time of *B. longum* are presented in Table 1. At the beginning of fermentation, the lactose content decreased rapidly but gradually decreased after 4 h of fermentation, and the difference according to the amount of *B. longum* inoculation was not significant. The lactose content ranged from 4.59% to 4.65% depending on the amount of *B. longum* inoculation before fermentation. After 2 h of fermentation, the percentage in the control group was 1.37% and ranged from 1.46 to 1.52%, depending on the amount of *B. longum* inoculation. After 8 h of fermentation, the lactose content was 0.29% in the control group and 0.39, 0.44, and 0.47% in the *B. longum* inoculation group. The lactose content did not change significantly depending on the presence or absence of *B. longum* inoculation, and these results were similar to those reported by Popescu et al. [19]. Considering that the domestic low-lactose yogurt standard is <0.5%, all treatments in this experiment were considered suitable.

### 3.3. pH, Total Acidity, and Lactic Acid Content

**Figure 1:**
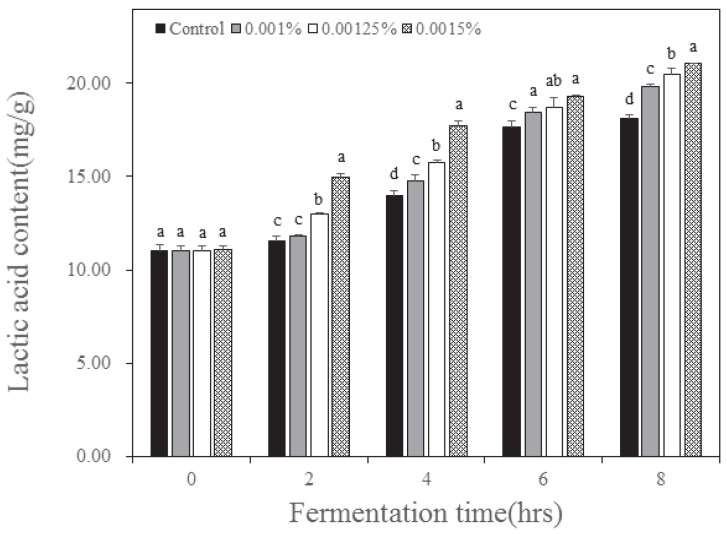
Change of lactic acid content in yogurt added with *Bifidobacterium longum* and lactase. Each value is expressed as the mean ±standard deviation (n = 3); means in the same fermentation time (a–d) are significantly different (*p* < 0.05) by Duncan’s multiple range test.

The pH of the yogurt according to the inoculation concentration and fermentation time of *B. longum* is presented in Table 2. As fermentation time increased, the pH of the yogurt decreased. The pH was 6.67 before fermentation, 8 h after fermentation the control group was at 4.69, and for *B. longum* inoculation concentrations of 0.001, 0.00125, and 0.0015% pH were 4.54, 4.47, and 4.42, respectively. As displayed in Table 2, the total acidity increased with fermentation time and increased concentration of *B. longum*. Total acidity was 0.10% before fermentation, and 8 h after fermentation the control was 0.59%. With increasing *B. longum* inoculation concentrations from 0.001 to 0.0015%, total acidity increased from 0.65 to 0.76%. The decrease in pH of yogurt and increase in total acidity according to *B. longum* inoculation concentration and fermentation time were similar to those reported by Irma et al. [20]. The lactic acid content displayed the same trend as total acidity (Figure 1). Similar to the total acidity, the lactic acid content increased with increasing concentrations of *B. longum*. Yogurt made by inoculating *B. longum* is known to increase the lactic acid content as fermentation time increases [21], and the same result was observed in this study. This change indicated that the increase in *B. longum* concentration can lead fermentation effectively, just like the change in sugar.

### 3.4. Viscosity

**Figure 2:**
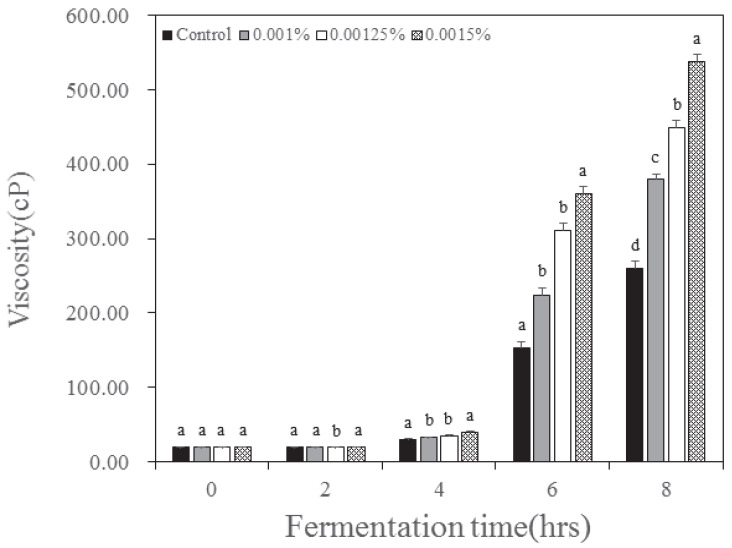
Change of viscosity in yogurt added with *Bifidobacterium longum* and lactase. Each value is expressed as the mean ± standard deviation (n = 3); means in the same fermentation time (a–d) are significantly different (*p* < 0.05) by Duncan’s multiple range test.

The viscosity of the yogurt according to the inoculation concentration and fermentation time of *B. longum* is depicted in Figure 2. The viscosity of the yogurt tended to increase with fermentation time, and the viscosity increased as the *B. longum* inoculation concentration increased. The control was increased from 19.89 cP before fermentation to 260.65 cP at the end of fermentation. The experimental group inoculated with 0.001% *B. longum* increased from 20.30 cP at the beginning to 380.13 cP at the end of fermentation. Additionally, the viscosity of the inoculation group with 0.00125% *B. longum* was increased to 448.67 and the inoculation group with 0.0015% was increased to 536.86 cP. Viscosity displayed the same trend as that of yogurt, which increased because of protein coagulation due to lowered pH during milk fermentation. Decreasing pH and disulfide bonds can lead to casein aggregation and result in gel formation [22]. These results are consistent with those of Yan et al. [7], who found that viscosity increased when *Lactobacillus bulgaricus*, *Streptococcus salivarius* subsp. *thermophilus*, and *Bifidobacterium longum* were used together. Additionally, base composition of milk, fermentation process, and starter culture can also affect the viscosity. Viscosity is one of the crucial texture properties of yogurt. In our result, the viscosity was the highest at the highest *B. longum* concentration. This may be due to interaction between exopolysaccharide (EPS) produced by *B. longum* and the milk protein, which affect the property of the gel structure in fermented milk and acidity.

### 3.5. Solids Non-Fat Content

**Figure 3:**
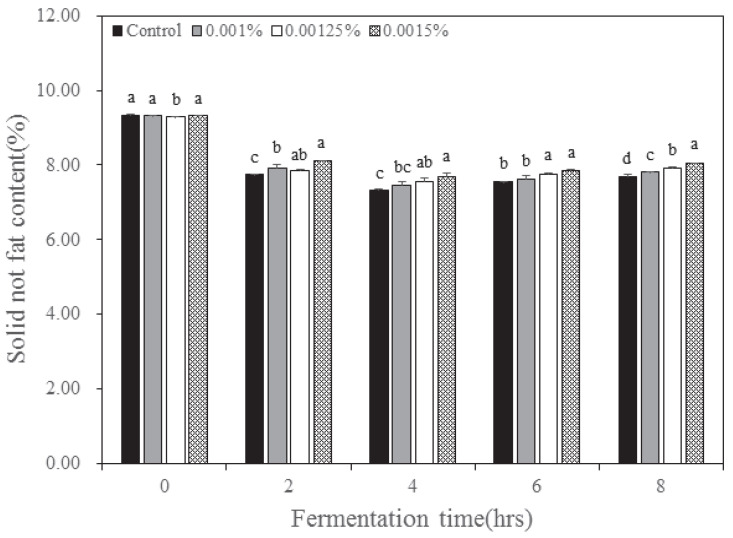
Change of solid not fat content in yogurt added with *Bifidobacterium longum* and lactase. Each value is expressed as the mean ± standard deviation (n = 3); means in the same fermentation time (a–d) are significantly different (*p* < 0.05) by Duncan’s multiple range test.

The solids non-fat content of yogurt according to the inoculation concentration and fermentation time of *B. longum* is illustrated in Figure 3. Solid non-fat content is the value obtained by subtracting fructose, glucose, sucrose, and crude fat from the solid content. The solid non-fat content decreased from 9.28–9.33% before fermentation to 7.68~8.04% after 8 h of fermentation, and the content was higher in 0.0015% of *B. longum* inoculation. In all experimental groups, the solid non-fat content displayed a tendency to decrease until 4 h of fermentation and then increase subsequently. The solid non-fat content increased because the activity of lactase was reduced by the pH and the conversion rate of glucose into acid increased, resulting in a decrease in glucose content. The experimental group that was inoculated with 0.0015% of *B. longum* displayed a solid non-fat content of 8% or more, which is the Korean standard for yogurt [23]. However, the control and inoculated group with 0.001% and 0.00125% of *B. longum* was unsuitable as per the Korean standard for yogurt because the solid non-fat content of the three group was less than 8%.

### 3.6. Total Lactic Acid Bacteria Cell Number

**Figure 4:**
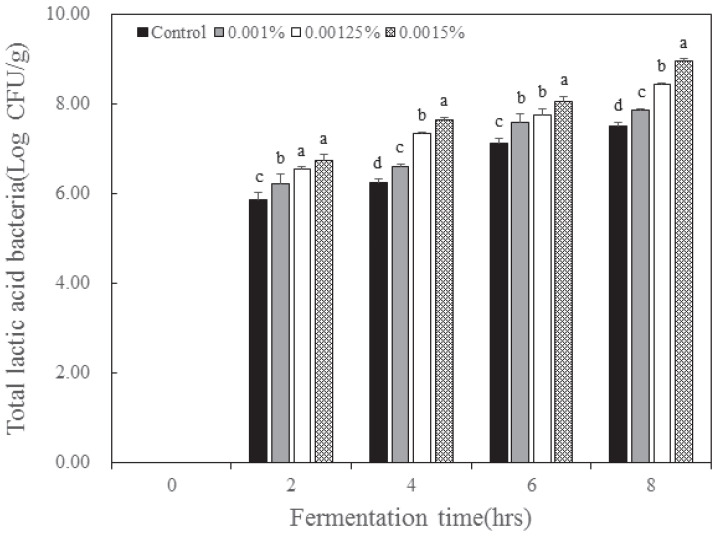
Change in total lactic acid bacteria count in yogurt added with *Bifidobacterium longum* and lactase. Each value is expressed as the mean ± standard deviation (n = 3); means in the same fermentation time (a–d) are significantly different (*p* < 0.05) by Duncan’s multiple range test.

The total number of lactic acid bacteria cells in the yogurt according to the inoculation concentration and fermentation time of *B. longum* is presented in Figure 4. Lactic acid bacteria were not detected in the sterilized milk. With increasing fermentation time, the groups inoculated with higher concentrations of *B. longum* displayed a higher number of total lactic acid bacteria. When *B. longum* was non-inoculated, the number of lactic acid bacteria after 8 h of fermentation was 7.49 log CFU/g, and when *B. longum* was inoculated at 0.001% concentration, the number of lactic acid bacteria after fermentation was 7.85 log CFU/g and was 8.44 log CFU/g at 0.00125% and 8.95 log CFU/g at 0.0015%, respectively. The control and 0.001% *B. longum* treatment groups indicated that the total number of lactic acid bacteria was less than 100 million, which is not suitable according to Korean yogurt standards. Additionally, the total lactic acid bacteria number of the 0.00125% and 0.0015% *B. longum* treatment groups was suitable according to Korean yogurt standards [23].

## 4. Conclusions

In this study, the fermentation and quality characteristics of yogurt were investigated based on the inoculation concentration of *Bifidobacterium longum*. As *B. longum* uses sugars to produce acids, the higher the concentration of *B. longum*, the faster the total sugar and reducing sugar content decreased. After fermentation, the lactose content ranged from 0.29 to 0.47% and was less than 0.5% in all experimental groups. The higher the concentration of *B. longum was*, the faster the sugar was converted to acid; therefore, when the amount of *B. longum* added was 0.0015%, the total acid and lactic acid contents were high in yogurt, and the number of total lactic acid bacteria and solid non-fat content were also high. From the above results, the experimental group inoculated with 0.0015% of *B. longum* had the best factors for determining the quality of yogurts, such as total acidity, pH, non-fat solids, and total lactic acid bacteria number compared to the other experimental groups.

## Figures and Tables

**Table 1:** Total sugar, reducing sugar, and lactose contents of yogurt added with *Bifidobacterium longum* and lactase.

*B. longum* Concentration (%)	Fermentation Time (h)	Total Sugar (mg/mL)	Reducing Sugar (mg/mL)	Lactose (%)
Control	0	79.42 ± 0.65 ^a (1) (2)^	44.33 ± 0.25 ^a^	4.65 ± 0.02 ^a^
	2	79.16 ± 0.24 ^a^	52.13 ± 0.84 ^a^	1.37 ± 0.05 ^b^
	4	78.63 ± 0.81 ^a^	53.89 ± 1.69 ^a^	0.42 ± 0.00 ^c^
	6	76.02 ± 0.37 ^a^	52.12 ± 0.18 ^a^	0.36 ± 0.00 ^d^
	8	73.98 ± 0.31 ^a^	50.63 ± 0.09 ^a^	0.29 ± 0.01 ^c^
0.001	0	79.54 ± 0.22 ^a^	44.67 ± 1.55 ^a^	4.62 ± 0.01 ^a^
	2	79.10 ± 0.38 ^a^	51.41 ± 0.12 ^ab^	1.46 ± 0.05 ^ab^
	4	78.08 ± 0.05 ^a^	52.10 ± 0.37 ^ab^	0.56 ± 0.00 ^b^
	6	74.81 ± 0.45 ^b^	50.43 ± 0.10 ^b^	0.48 ± 0.01 ^c^
	8	72.61 ± 0.48 ^b^	48.93 ± 0.17 ^b^	0.39 ± 0.01 ^b^
0.00125	0	79.17 ± 0.42 ^a^	44.49 ± 1.21 ^a^	4.59 ± 0.01 ^a^
	2	78.97 ± 0.06 ^a^	49.93 ± 0.79 ^bc^	1.50 ± 0.07 ^a^
	4	77.48 ± 1.08 ^a^	51.44 ± 1.07 ^b^	0.59 ± 0.04 ^b^
	6	73.11 ± 0.65 ^c^	50.12 ± 0.47 ^b^	0.52 ± 0.02 ^b^
	8	71.40 ± 0.29 ^c^	47.95 ± 0.70 ^bc^	0.44 ± 0.02 ^a^
0.0015	0	79.42 ± 1.25 ^a^	44.12 ± 0.05 ^a^	4.62 ± 0.17 ^a^
	2	78.50 ± 1.25 ^a^	48.54 ± 1.65 ^c^	1.52 ± 0.01 ^a^
	4	76.14 ± 0.27 ^b^	50.19 ± 0.36 ^b^	0.62 ± 0.02 ^a^
	6	71.67 ± 0.11 ^d^	48.38 ± 0.89 ^c^	0.55 ± 0.02 ^a^
	8	69.91 ± 0.70 ^d^	46.97 ± 0.79 ^c^	0.47 ± 0.01 ^a^

(1) Values are Mean ± SD (n = 3). (2) Different small letters (a–d) in the same column indicate a significant difference by Duncan’s range test (*p* < 0.05).

**Table 2:** pH and total acidity in yogurt added with *Bifidobacterium longum* and lactase.

*B. longum* Concentration (%)	Fermentation Time (h)	pH	Total Acidity (%)
Control	0	6.67 ± 0.01 ^a (1) (2)^	0.10 ± 0.00 ^a^
	2	6.61 ± 0.02 ^a^	0.11 ± 0.00 ^b^
	4	5.84 ± 0.01 ^a^	0.19 ± 0.01 ^c^
	6	4.99 ± 0.01 ^a^	0.44 ± 0.01 ^d^
	8	4.69 ± 0.01 ^a^	0.59 ± 0.01 ^d^
0.001	0	6.67 ± 0.02 ^a^	0.10 ± 0.01 ^a^
	2	6.57 ± 0.02 ^b^	0.11 ± 0.00 ^b^
	4	5.69 ± 0.05 ^b^	0.22 ± 0.00 ^b^
	6	4.88 ± 0.05 ^b^	0.49 ± 0.00 ^c^
	8	4.69 ± 0.01 ^b^	0.65 ± 0.00 ^c^
0.00125	0	6.68 ± 0.01 ^a^	0.10 ± 0.01 ^a^
	2	6.49 ± 0.01 ^c^	0.16 ± 0.01 ^a^
	4	5.53 ± 0.01 ^c^	0.26 ± 0.00 ^a^
	6	4.84 ± 0.01 ^bc^	0.52 ± 0.01 ^b^
	8	4.47 ± 0.01 ^c^	0.68 ± 0.01 ^b^
0.0015	0	6.68 ± 0.01 ^a^	0.10 ± 0.01 ^a^
	2	6.49 ± 0.01 ^c^	0.16 ± 0.01 ^a^
	4	5.53 ± 0.01 ^c^	0.26 ± 0.02 ^a^
	6	4.80 ± 0.01 ^c^	0.58 ± 0.01 ^a^
	8	4.42 ± 0.02 ^c^	0.76 ± 0.01 ^a^

(1) Values are mean ± SD (n = 3). (2) Different small letters (a–d) in the same column indicate a significant difference by Duncan’s range test (*p* < 0.05).
